# Risk factors for failed extubation within 7 days in elderly critically ill patients based on respiratory mechanics and clinical indicators: a retrospective cohort study

**DOI:** 10.3389/fmed.2025.1721952

**Published:** 2025-11-19

**Authors:** Xixi Ruan, Xiaping Zhang, Weihang Hu, Liang Wu, Yikuan Shen

**Affiliations:** 1 Department of Critical Care Medicine, Zhejiang Hospital, Hangzhou, China; 2 Department of Acupuncture, Xinchang Hospital of Chinese Medicine, Xinchang, China

**Keywords:** elderly critical illness, tracheal extubation, extubation failure, risk factor, respiratory mechanics

## Abstract

**Background:**

Tracheal intubation and mechanical ventilation are common treatments for critically ill patients. Extubation failure is associated with prolonged mechanical ventilation, increased incidence of ventilator-associated pneumonia (VAP), and elevated mortality. Elderly patients face unique challenges. Thus, early risk assessment for weaning and extubation in elderly patients is crucial. This study aimed to identify independent risk factors for extubation failure within 7 days in elderly critically ill patients.

**Methods:**

A retrospective cohort study was conducted on 103 elderly patients with tracheal intubation admitted to our ICU from January 2022 to July 2025. Clinical characteristics of patients with extubation failure within 7 days were analyzed. The optimal cutoff values for continuous variables were determined using the Youden index. Logistic regression analysis was performed to identify independent risk factors for extubation failure within 7 days.

**Results:**

There was a high rate of extubation failure within 7 days in elderly critically ill patients. Logistic regression showed that female (OR = 6.770, 95% CI 1.100–41.654, *p* = 0.039), serum sodium >145.76 mmol/L (OR = 15.008, 95% CI 1.189–189.465, *p* = 0.036), lactate >3.45 mmol/L (OR = 17.866, 95% CI 1.907–167.378, *p* = 0.012), and peak airway pressure (Ppeak) > 18.5 cmH₂O (OR = 9.056, 95% CI 1.705–48.111, *p* = 0.010) were positively correlated with extubation failure, serving as independent risk factors.

**Conclusion:**

Female, serum sodium >145.76 mmol/L, lactate >3.45 mmol/L at intubation, and Ppeak >18.5 cmH₂O at relative stability after intubation are independent risk factors for extubation failure within 7 days in elderly patients with tracheal intubation.

## 1 Introduction

Tracheal intubation and mechanical ventilation are essential treatment for critically ill patients but carry risks of complications such as catheter displacement, airway injury, artificial airway obstruction, and bleeding (1). Prolonged mechanical ventilation increases the complication rates, lengthens hospital stays, impairs pulmonary function, and may cause neuropsychiatric complications (2, 3). Therefore, timely weaning and extubation are critical to reduce complications and promote early recovery. The average duration of tracheal intubation in critically ill patients is 2–8 days (4), but elderly patients are at higher risk of weaning difficulties due to age-related physiological decline, frailty, weakened cough/swallowing reflexes, and increased susceptibility to respiratory failure (5). Thus, risk assessment for early extubation in elderly patients is of great clinical importance.

Extubation failure was linked to multiple factors, including age, primary disease, airway anatomical abnormalities, laryngeal edema, frailty, high risk of aspiration, and internal environment disorders (6, 7). It was associated with prolonged mechanical ventilation, higher VAP incidence, and increased mortality (8). For patients with weaning difficulties, tracheostomy within 1 week has been shown to reduce sedative use, lower the incidence of ventilator-associated pneumonia (VAP), and shorten intensive care unit (ICU) stays (9). Identifying risk factors for extubation failure within 7 days in elderly critically ill patients can guide early risk assessment, optimize tracheostomy timing, reduce risks from prolonged intubation, improve prognosis, shorten hospital stays, and reduce healthcare resource consumption, which holding significant clinical value.

## 2 Materials and methods

### 2.1 Study design and patients

This was a single-center retrospective cohort study. The hospital is a provincial hospital. The number of ICU beds is 25, and the average bed utilization rate is about 85%. We included patients admitted to the ICU of our hospital from January 2022 to July 2025 who met the inclusion criteria and excluded those with exclusion criteria. Extubation failure was defined as the need for reintubation, initiation of non-invasive ventilation, or death within 48 h after planned extubation (7). Patients were divided into the successful extubation group and failed extubation group based on outcomes within 7 days after intubation.

Inclusion criteria: 1. Aged ≥65 years; 2. ICU stay ≥48 h. Exclusion criteria: 1. Incomplete clinical data; 2. Pre-admission intubation duration >24 h; 3. Planned tracheostomy within 48 h after intubation.

### 2.2 Data collection

Clinical data were retrospectively collected from medical records, including:

Baseline characteristics: Sex, age, smoking history, comorbidities [chronic kidney disease, cardiovascular disease, liver disease, chronic obstructive pulmonary disease (COPD), bronchiectasis]; Disease-related data: Causes of intubation, Acute Physiology and Chronic Health Evaluation II (APACHE II) score, Glasgow Coma Scale (GCS) score before intubation; vital signs (temperature, blood pressure, heart rate, oxygen saturation) during relative stability after intubation; Laboratory test results: Arterial blood gas analysis after intubation, routine blood tests, C-reactive protein (CRP), procalcitonin (PCT), liver/kidney function, electrolytes, and B-type natriuretic peptide (BNP) after intubation; Respiratory mechanics parameters during relative stability after intubation: Fraction of inspired oxygen (FiO₂), respiratory rate (RR), tidal volume (VT), positive end-expiratory pressure (PEEP), plateau pressure (Pplat), dynamic compliance (Cydn), airway resistance (RAW), peak airway pressure (Ppeak), and ventilator mode [Bi-level Positive Airway Pressure (BIPAP), Continuous Positive Airway Pressure (CPAP), Pressure Control (PC), Synchronized Intermittent Mandatory Ventilation (SIMV)].

### 2.3 Statistical analysis

Categorical variables were described as frequencies (percentages) and compared using the chi-square test or Fisher’s exact test (for small samples). Continuous variables were expressed as mean (standard) deviation (normally distributed) or median (25th, 75th percentiles) (non-normally distributed), with comparisons using independent samples *t*-test or Mann–Whitney *U* test, respectively.

For continuous variables with statistical significance, receiver operating characteristic (ROC) curves and the Youden index were used to determine optimal cutoff values, which were then converted to binary variables. Variables with *p* < 0.05 in univariate Logistic regression were included in multivariate Logistic regression to identify independent risk factors for extubation failure within 7 days.

All tests were two-tailed, with *p* < 0.05 considered statistically significant. Data analysis was performed using SPSS 27.0, and graphs were generated using R 4.3.0.

During the entire data collection and analysis process, none of the authors had access to any information that could identify individual participants. This included the exclusion of direct identifiers (e.g., full name, national identification number, contact information) and indirect identifiers (e.g., specific date of birth, unique clinical record numbers) from the dataset provided for this study. The data were pre-processed and de-identified prior to access, ensuring full protection of participant privacy.

## 3 Results

### 3.1 Baseline characteristics of patients

A total of 103 patients were included, with 22 in the successful extubation group and 81 in the failed extubation group.

Sex and age: The proportion of males in the failed extubation group was significantly lower than in the successful extubation group (58.02% vs. 81.82%, χ^2^ = 4.207, *p* = 0.040), and the failed extubation group was older (90.00 [78.00, 94.00] years vs. 82.32 ± 9.31 years, *Z* = −2.066, *p* = 0.039).

Other baseline factors: No significant differences were observed in smoking history, comorbidities, APACHE II score, GCS score, or causes of intubation between the two groups (all *p* > 0.05) (Table 1).

**Table 1:** Baseline characteristics of patients with successful and failed extubation within 7 days.

	Successful extubation (*n* = 22)	Failed extubation (*n* = 81)	*t/Z/* $\chi^{2}$	*p*
Gender			**4.207**	**0.040**
Male gender (*n*, %)	**18.00 (81.82%)**	**47.00 (58.02%)**		
Female gender (*n*, %)	**4.00 (18.18%)**	**34 (41.98%)**		
Age (years)	**82.32 (9.31)**	**90.00 (78.00, 94.00)**	**−2.066**	**0.039**
Smoking history (*n*, %)	6.00 (27.27%)	14.00 (17.28%)	#	0.362
Reasons for tracheal intubation			#	0.099
Respiratory failure (*n*, %)	19.00 (86.36%)	54.00 (66.67%)		
Airway protection (*n*, %)	1.00 (4.55%)	3.00 (3.70%)		
Circulatory failure (*n*, %)	2.00 (9.09%)	24.00 (29.63%)		
Comorbidities				
Chronic kidney disease (*n*, %)	5.00 (22.73%)	23.00 (28.40%)	0.281	0.596
Chronic cardiovascular disease (*n*, %)	6.00 (27.27%)	36.00 (44.44%)	2.111	0.146
Chronic liver disease (*n*, %)	2.00 (9.09%)	10.00 (12.35%)	#	1.000
COPD (*n*, %)	1.00 (4.54%)	7.00 (8.64%)	#	1.000
Bronchiectasis (*n*, %)	1.00 (4.54%)	2.00 (2.47%)	#	0.518
APACHE II score at enrollment	24.73 (8.44)	25.58 (7.10)	−0.480	0.633
GCS score at enrollment	8.00 (6.00, 10.00)	9.00 (5.00, 12.00)	−0.433	0.665

#: Fisher’s exact test was used; COPD, chronic obstructive pulmonary disease; APACHE II, acute physiology and chronic health evaluation II; GCS, Glasgow Coma Scale.The bold values represent statistical differences in this measure.

### 3.2 Clinical manifestations and laboratory findings

Vital signs: systolic blood pressure (SBP) (106.00 [95.00, 131.00]mmHg vs. 131.95 ± 25.85 mmHg, *Z* = −2.930, *p* = 0.003), diastolic blood pressure (DBP) (60.00 [46.00, 68.00]mmHg vs. 75.50 ± 23.11 mmHg, *Z* = −2.918, *p* = 0.004), and mean arterial pressure (MAP) (75.00 [65.00, 86.00]mmHg vs. 94.36 ± 20.42 mmHg, *Z* = −3.200, *p* = 0.001) in the failed extubation group were significantly lower than in the successful extubation group (all *p* < 0.01), while temperature, pulse pressure, heart rate, and oxygen saturation showed no differences (all *p* > 0.05) (Table 2).

**Table 2:** Clinical manifestations and laboratory findings after intubation.

	Successful extubation (*n* = 22)	Failed extubation (*n* = 81)	*t/Z*	*p*
Vital signs				
Body temperature (°C)	36.78 (0.86)	36.89 (1.16)	−0.404	0.687
SBP (mmHg)	**131.95 (25.85)**	**106.00 (95.00, 131.00)**	**−2.930**	**0.003**
DBP (mmHg)	**75.50 (23.11)**	**60.00 (46.00, 68.00)**	**−2.918**	**0.004**
Pulse pressure (mmHg)	62.00 (31.00, 79.00)	48 (40.00, 65.00)	−1.107	0.268
MAP (mmHg)	**94.36 (20.42)**	**75.00 (65.00, 86.00)**	**−3.200**	**0.001**
HR (bpm)	98.73 (20.46)	105.21 (25.29)	−1.107	0.271
SPO_2_ (%)	100.00 (99.00, 100.00)	100.00 (98.00, 100.00)	−0.743	0.457
Laboratory results				
White blood cell count (*10^9^/L)	11.42 (5.49)	12.40 (8.50, 17.42)	−1.219	0.223
Neutrophilic percentage (%)	88.30 (81.45, 93.00)	89.20 (81.60, 92.90)	−0.402	0.687
Lymphocyte count (*10^9^/L)	0.75 (0.31, 1.08)	0.70 (0.40, 1.47)	−0.109	0.913
Hemoglobin concentration (g/L)	97.18 (31.29)	96.33 (25.83)	0.130	0.896
Platelet count (*10^9^/L)	188.00 (148.00, 231.00)	158.22 (77.96)	−1.316	0.188
CRP (mg/L)	47.88 (19.84, 93.44)	62.49 (22.63 28.60)	−0.978	0.328
PCT (ng/L)	2.36 (0.26, 3.97)	1.0.52 (0.54, 4.56)	−0.089	0.929
Albumin (g/L)	31.63 (3.17)	30.48 (4.20)	1.194	0.235
Total protein (g/L)	58.91 (55.78, 63.40)	57.16 (7.50)	−1.006	0.314
ALT (U/L)	30.00 (19.00, 42.00)	19.00 (12.00, 56.00)	−0.539	0.590
AST (U/L)	49.00 (23.00, 59.00)	39.00 (22.00, 123.00)	−0.394	0.693
Scr ( μ mol/L)	100.5 (76.00, 183.00)	130.00 (95.00, 209.00)	−1.432	0.152
BUN (mmol/L)	**12.52 (6.73, 19.01)**	**16.69 (11.76, 27.39)**	**−2.567**	**0.010**
GFR (mL/min/1.73m^2^)	56.75 (30.04)	37.80 (20.30, 62.70)	−1.963	0.050
Serum sodium (mmol/L)	**139.67 (5.29)**	**144.43 (8.57)**	**−2.257**	**0.024**
Serum potassium (mmol/L)	4.26 (3.60, 4.65)	4.31 (3.94, 4.69)	−0.608	0.543
BNP (pg/mL)	519.00 (250.00, 776.00)	360.00 (198.00, 705.00)	−0.933	0.351

HR, heart rate; SBP, systolic blood pressure; DBP, diastolic blood pressure; MAP, mean arterial pressure; WBC, white blood cell count; CRP, C-reactive protein; PCT, procalcitonin; ALT, alanine transaminase; AST, aspartate transaminase; Scr, serum creatinine; BUN, blood urea nitrogen; GFR, glomerular filtration rate; BNP, B-type natriuretic peptide.The bold values represent statistical differences in this measure.

Laboratory results: Urea (16.69 [11.76, 27.93] vs. 12.52 [6.73, 19.01] mmol/L, *Z* = −2.576, *p* = 0.010) and serum sodium (144.43 ± 8.57 vs. 139.67 ± 5.29 mmol/L, *Z* = −2.257, *p* = 0.024) were significantly higher in the failed extubation group. No differences were observed in other indices (all *p* > 0.05) (Table 2).

### 3.3 Blood gas analysis and respiratory mechanics

Blood gas indices: Lactate levels in the failed extubation group were significantly higher (2.76 [1.60, 8.60] vs. 1.80 [1.10, 2.66] mmol/L, *Z* = −2.745, *p* = 0.006), with no differences in pH, PaO₂, PaCO₂, or PaO₂/FiO₂ (all *p* > 0.05) (Table 3).

**Table 3:** Blood gas analysis and respiratory mechanics parameters.

	Successful extubation (*n* = 22)	Failed extubation (*n* = 81)	*t*/*z*/ $\chi^{2}$	*p*
Blood gas analysis				
PH	7.37 (7.30, 7.43)	7.40 (7.26, 7.48)	−0.519	0.604
PaO_2_ (mmHg)	131.00 (88.00, 190.00)	125.00 (80.00, 193.00)	−0.113	0.910
PaCO_2_ (mmHg)	38.50 (34.00, 57.00)	39.00 (31.00, 47.00)	−0.568	0.570
BE (mmol/L)	4.85 (2.60, 6.50)	5.90 (3.70, 11.40)	−1.42	0.155
Lac (mmol/L)	**1.80 (1.10, 2.66)**	**2.76 (1.60, 8.60)**	**−2.745**	**0.006**
FiO_2_ (%)	50.00 (45.00, 60.00)	55.00 (45.00, 65.00)	−1.030	0.303
PaO_2_/FiO_2_ (mmHg)	281.50 (143.30)	227.00 (152.00, 372.00)	−0.390	0.696
Respiratory mechanics				
RR (bpm)	**15.00 (14.00, 18.00)**	**16.00 (15.00, 20.00)**	**−1.972**	**0.049**
VT (mL)	485.27 (89.46)	460.00 (390.00, 556.00)	−0.688	0.491
PEEP (cmH_2_O)	5.00 (4.00, 8.00)	5.00 (5.00, 6.00)	−0.396	0.692
Pplat (cmH_2_O)	18.00 (16.00, 22.00)	20.00 (18.00, 23.00)	−1.792	0.073
RAW (cmH_2_O*s/L)	14.00 (12.00, 19.00)	15.60 (12.60, 20.00)	−0.833	0.405
Cydn(mL/cmH_2_O)	**37.35 (20.00, 54.00)**	**26.00 (20.00, 42.30)**	**−2.503**	**0.012**
Ppeak (cmH_2_O)	**19.45 (3.60)**	**21.00 (19.00, 24.00)**	**−2.016**	**0.044**
Ventilator mode				
BIPAP (*n*, %)	21.00 (95.55%)	73.00 (90.13%)	#	1.000
CPAP (*n*, %)	0.00 (0.00%)	3.00 (3.70%)		
PC (*n*, %)	1.00 (4.45%)	3.00 (3.70%)		
SIMV (*n*, %)	0.00 (0.00%)	2.00 (2.47%)		

#: Fisher’s exact test was used. BE, base excess; FiO₂, fraction of inspired oxygen; RR, respiratory rate; VT, tidal volume; PEEP, positive end-expiratory pressure; Pplat, plateau pressure; RAW, airway resistance; Cydn, dynamic compliance; Ppeak, peak airway pressure; BIPAP, bi-level positive airway pressure; CPAP, continuous positive airway pressure; PC, pressure control; SIMV, synchronized intermittent mandatory ventilation.The bold values represent statistical differences in this measure.

Respiratory mechanics: The failed extubation group had higher RR (16.00 [15.00, 20.00] vs. 15.00 [14.00, 18.00] bpm, *Z* = −1.972, *p* = 0.049) and Ppeak (21.00 [19.00, 24.00] vs. 19.45 ± 3.60 cmH₂O, *Z* = −2.016, *p* = 0.044), and lower Cydn (26.00 [20.00, 42.30] vs. 37.35 [20.00, 54.00] mL/cmH₂O, *Z* = −2.503, *p* = 0.012). Ventilator modes, Pplat, RAW, RR and PEEP showed no significant difference (*p* > 0.05) (Table 3).

### 3.4 Optimal cutoff values of continuous variables

**Figure 1:**
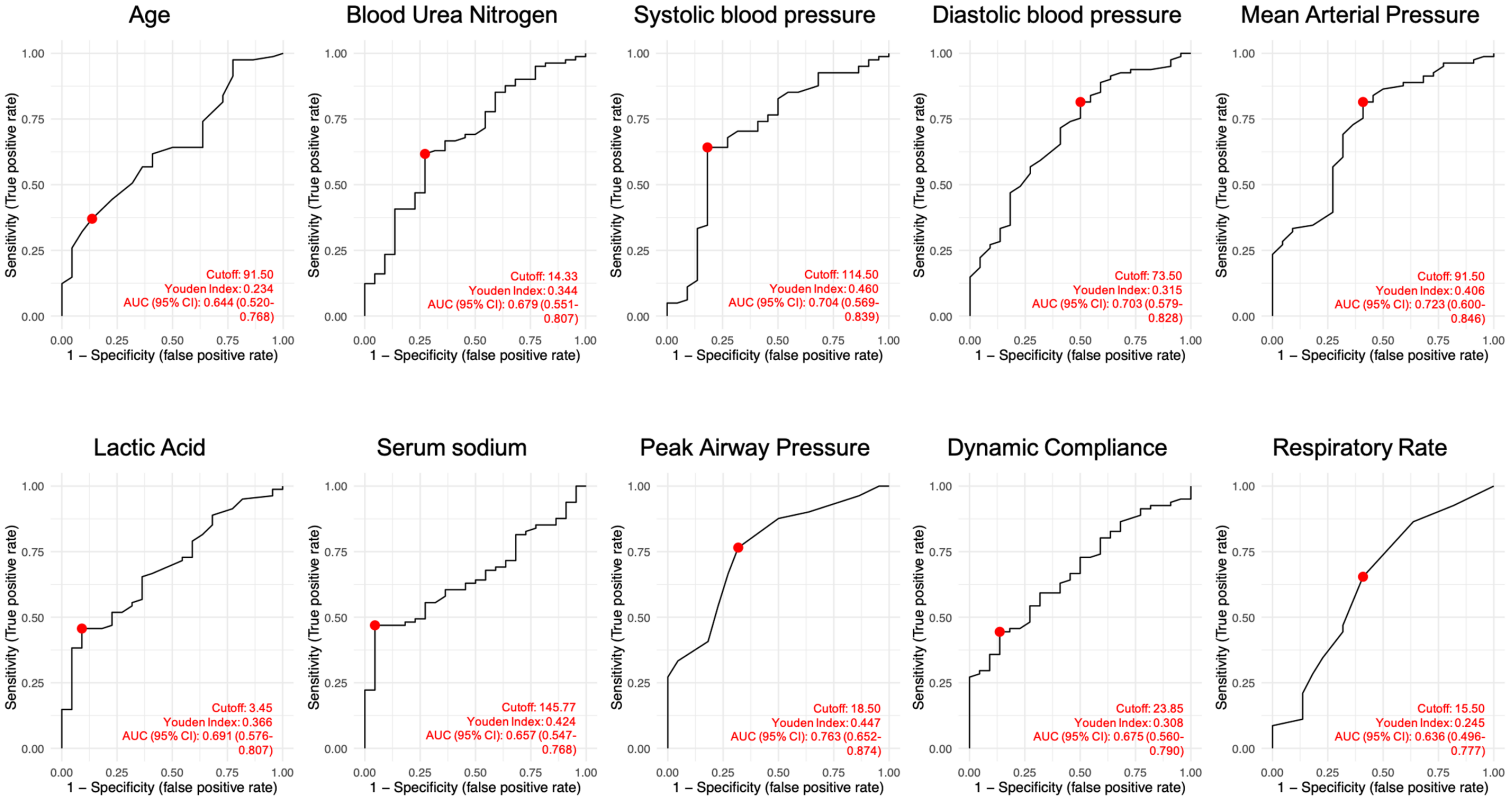
ROC curves for statistically significant continuous variables (Youden index optimal method).

ROC curves showed that continuous variables with statistical significance had predictive value for extubation failure. Using the Youden index, optimal cutoff values were determined as follows: age 91.5 years, SBP 114.5 mmHg, DBP 73.5 mmHg, MAP 91.5 mmHg, BUN 14.33 mmol/L, serum sodium 145.77 mmol/L, lactate 3.45 mmol/L, RR 15.5 bpm, Cydn 23.85 mL/cmH₂O, and Ppeak 18.5 cmH₂O (Table 4 and Figure 1).

**Table 4:** Cutoff values of statistically significant continuous variables.

	Best cut-off value	Youden index	Sensitivity	Specificity	AUC (95% CI)
Age (years)	91.50 (>91.5 years)	0.234	0.370	0.864	0.644 (0.520–0.768)
SBP (mmHg)	114.50 (>114.5 mmHg)	0.460	0.642	0.818	0.704 (0.569–0.839)
DBP (mmHg)	73.50 (>73.5 mmHg)	0.315	0.815	0.500	0.703 (0.579–0.828)
MAP (mmHg)	91.5 (>91.5 mmHg)	0.406	0.815	0.591	0.723 (0.600–0.846)
BUN (mmol/L)	14.33 (>14.33 mmol/L)	0.345	0.617	0.727	0.679 (0.551–0.807)
Serum sodium (mmol/L)	145.77 (>145.76 mmol/L)	0.424	0.469	0.955	0.657 (0.547–0.768)
Lac (mmol/L)	3.45 (>3.45 mmol/L)	0.366	0.457	0.909	0.691 (0.576–0.807)
RR (bpm)	15.50 (>15.5 bpm)	0.245	0.654	0.591	0.636 (0.496–0.777)
Cydn (mL/cmH₂O)	23.85 (>23.85 mL/cmH₂O)	0.308	0.444	0.854	0.675 (0.560–0.790)
Ppeak (cmH_2_O)	18.50 (>18.5 cmH_2_O)	0.447	0.765	0.682	0.763 (0.652–0.874)

The bold values represent statistical differences in this measure.

### 3.5 Risk factors associated with Extubation failure

Univariate Logistic regression showed that, except Cydn > 23.85 mL/cmH_2_O (*p* = 0.072), female, age > 91.5 years old, SBP > 114.5 mmHg, DBP > 73.5 mmHg, MAP > 91.5 mmHg, BUN > 14.33 mmol/L, Serum sodium > 145.76 mmol/L, Lac > 3.45 mmol/L, RR > 15.5 bpm and Ppeak > 18.5cmH_2_O were related to extubation failure (all *p* < 0.05).

Multivariate Logistic regression identified four independent risk factors for extubation failure within 7 days: Female sex (OR = 6.770, 95% CI 1.100–41.654, *p* = 0.039); Serum sodium >145.76 mmol/L (OR = 15.008, 95% CI 1.189–189.465, *p* = 0.036); Lactate >3.45 mmol/L (OR = 17.866, 95% CI 1.907–167.378, *p* = 0.012); Ppeak >18.5 cmH₂O (OR = 9.056, 95% CI 1.705–48.111, *p* = 0.010) (Table 5).

**Table 5:** Univariate and multivariate logistic regression analysis of risk factors for extubation failure.

	Univariate logistic regression analysis		Multivariate logistic regression analysis	
	OR (95% CI)	*p*	OR (95% CI)	*p*
Female gender	**3.255 (1.011–41.654)**	**0.048**	**6.770 (1.100–41.654)**	**0.039**
Age > 91.5 years	3.725 (1.017–13.649)	0.047	2.694 (0.380–19.080)	0.321
SBP > 114.5 mmHg	0.117 (0.036–0.381)	<0.001	0.246 (0.036–1.698)	0.155
DBP > 73.5 mmHg	0.227 (0.038–0.622)	0.004	0.779 (0.046–27.776)	0.863
MAP > 91.5 mmHg	0.157 (0.057–0.436)	<0.001	1.163 (0.049–27.776)	0.926
BUN > 14.33 mmol/L	4.301 (1.521–12.166)	0.005	2.943 (0.522–16.583)	0.221
Serum sodium > 145.76 mmol/L	**18.558 (2.382–144.591)**	**0.005**	**15.008 (1.189–189.465)**	**0.036**
Lac > 3.45 mmol/L	**8.409 (1.843–28.366)**	**0.006**	**17.866 (1.907–167.378)**	**0.012**
RR > 15.5 bpm	2.734 (1.041–7.179)	0.041	0.856 (0.179–4.084)	0.845
Cydn>23.85 mL/cmH₂O	0.368 (0.124–1.093)	0.072	/	/
Ppeak>18.5 cmH_2_O	**4.713 (1.746–12.726)**	**0.002**	**9.056 (1.705–48.111)**	**0.010**

The bold values represent statistical differences in this measure.

## 4 Discussion

Extubation failure is associated with poor outcomes in critically ill patients, including mortality rates ranging from 25 to 50% (10). Current research on extubation primarily focuses on the entire treatment course, with limited data on early extubation risk factors, especially in non-surgical patients.

This study found that female is an independent risk factor for early extubation failure in elderly critically ill patients. Females have smaller central airway lumen and are more rely on accessory inspiratory muscles such as scalenus muscle (11). Aging causes muscle loss reduces respiratory muscle mass and strength, particularly in elderly females (12), leading to decreased chest wall compliance, reduced expiratory flow, and limited upper lung joint mobility, which impair pulmonary function (13). Additionally, postmenopausal estrogen decline may weaken metabolic reflex of respiratory muscles (11). These factors likely contribute to higher extubation failure risk in elderly females due to a more significant decline in lung function.

Lactate is an important indicator in the ICU, elevated lactate is a marker of poor prognosis in ICU patients (14). We found that lactate is also an independent risk factor for early extubation failure in elderly critically ill patients. Lactate elevation may result from hypoxia (e.g., due to septic/cardiogenic/hypovolemic shock), liver disease, medication use (e.g., metformin), toxins, or trauma (15). In elderly critically ill patients, such underlying conditions are often life-threatening, suggesting that the primary condition is critical and may lead to an increased risk of early extubation failure.

Hypernatremia (serum sodium >145.76 mmol/L) was associated with higher extubation failure risk. Hypernatremia has adverse effects on a variety of physiological functions and is an independent risk factor for increased mortality in critically ill patients (16). Hypernatremia can lead to increased osmotic pressure of extracellular fluid, interfere with the intracellular metabolic environment (such as enzyme activity and ion gradient), and may affect the synthesis and release of neurotransmitters. At the same time, brain dehydration caused by hypernatremia can cause severe central nervous system dysfunction, interfere with the regulatory signal transmission of muscle by motor cortex, and may lead to decreased coordination of muscle activity and abnormal tension (such as rigidity or relaxation), affecting neuromuscular function (17). Therefore, we speculated that hypernatremia may affect the metabolism of respiratory center and respiratory muscle, resulting in decreased coordination and abnormal tone of respiratory muscle, thereby affecting lung function and increasing the risk of early extubation failure.

Ppeak is the pressure obtained when air is pushed into the lungs during inspiration and is a measure of airway resistance (18). Peak airway pressure is affected by airway resistance, lung compliance, tidal volume and inspiratory flow. Elevated Ppeak may indicate increased airway resistance (e.g., bronchospasm, secretion obstruction) or decreased lung compliance (e.g., pulmonary edema, pulmonary fibrosis) (19). Impaired respiratory function increases the risk of extubation (20). We speculated that the increase of peak airway pressure may indicate high airway resistance, decreased compliance, and poor basic pulmonary function. In addition, high peak airway pressure may increase the risk of secondary lung injury. A variety of reasons together lead to a high rate of early extubation failure. Although there was no statistical difference in Pplat between the two groups in this study, *p* = 0.073 was borderline significant. In the future, the interaction between Pplat and Ppeak can be explored by expanding the sample size.

This study was a retrospective cohort study and has certain limitations. First, by design, the majority of patients who had undergone surgery were not enrolled. Second, this study was a single-center study, which may have selection bias. Third, the sample size included in this study was limited, which may have masked associations (e.g., GFR, *p* = 0.050; intubation due to circulatory failure, *p* = 0.099). Forth, l ong-term outcomes such as post-ICU mortality or functional recovery can be assessed with subsequent long-term follow-up. Therefore, the results of this study need to be further verified in multicenter prospective cohort studies. In the future, prospective multicenter cohort studies can be carried out in multiple hospitals in the province to confirm the stability of the four independent risk factors found in this study.

## 5 Conclusion

This study found that the failure rate of early extubation in elderly patients was as high as 78.6%. Female, serum sodium >145.76 mmol/L, lactate >3.45 mmol/L at intubation, and Ppeak >18.5 cmH₂O during relative stability after intubation are independent risk factors for extubation failure within 7 days in elderly critically ill patients. Clinical management should focus on respiratory muscle training, correcting electrolyte disturbances (e.g., hypernatremia), optimizing airway care, and assessing tracheostomy timing in high-risk patients to improve outcomes. And future prospective multicenter studies are warranted to validate these findings and develop an elderly-specific extubation risk score.

## Data Availability

The original contributions presented in the study are included in the article/supplementary material, further inquiries can be directed to the corresponding author.

## Glossary

VAP: ventilator-associated pneumonia
ICU: intensive care unit
COPD: chronic obstructive pulmonary disease
APACHE II: acute physiology and chronic health evaluation II
GCS: Glasgow Coma Scale
ROC: receiver operating characteristic
SBP: systolic blood pressure
DBP: diastolic blood pressure
MAP: mean arterial pressure
WBC: white blood cell count
HR: heart rate
SPO_2_: pulse blood oxygen saturation
CRP: C-reactive protein
PCT: procalcitonin
BNP: brain natriuretic peptide
AST: aspartate aminotransferase
ALT: alanine aminotransferase
Scr: serum creatinine
BUN: blood urea nitrogen
GFR: glomerular filtration rate
PH: potential of hydrogen
BE: base excess
Lac: lactic acid
RR: respiratory rate
VT: tidal volume
PEEP: positive end-expiratory pressure
Pplat: plateau pressure
RAW: airway resistance
Cydn: dynamic compliance
Ppeak: peak airway pressure
BIPAP: bi-level positive airway pressure
CPAP: continuous positive airway pressure
PC: pressure control
SIMV: synchronized intermittent mandatory ventilation
